# Novel renal medullary carcinoma cell lines, UOK353 and UOK360, provide preclinical tools to identify new therapeutic treatments

**DOI:** 10.1002/gcc.22847

**Published:** 2020-04-17

**Authors:** Darmood Wei, Youfeng Yang, Christopher J. Ricketts, Cathy D. Vocke, Mark W. Ball, Carole Sourbier, Darawalee Wangsa, Danny Wangsa, Rajarshi Guha, Xiaohu Zhang, Kelli Wilson, Lu Chen, Paul S. Meltzer, Thomas Ried, Craig J. Thomas, Maria J. Merino, W. Marston Linehan

**Affiliations:** ^1^ Urologic Oncology Branch, Center for Cancer Research National Cancer Institute, National Institutes of Health Bethesda Maryland United States; ^2^ Genetics Branch, Center for Cancer Research National Cancer Institute, National Institutes of Health Bethesda Maryland United States; ^3^ Division of Preclinical Innovation, National Center for Advancing Translational Sciences National Institutes of Health Rockville Maryland United States; ^4^ Laboratory of Pathology National Cancer Institute, National Institutes of Health Bethesda Maryland United States

**Keywords:** Bortezomib, EZH2 inhibitor, INI1, renal medullary carcinoma, RMC, SMARCB1, SWI/SNF complex, UOK353, UOK360

## Abstract

Renal medullary carcinoma (RMC) is a rare, aggressive disease that predominantly afflicts individuals of African or Mediterranean descent with sickle cell trait. RMC comprises 1% of all renal cell carcinoma diagnoses with a median overall survival of 13 months. Patients are typically young (median age—22) and male (male:female ratio of 2:1) and tumors are characterized by complete loss of expression of the SMARCB1 tumor suppressor protein. Due to the low incidence of RMC and the disease's aggressiveness, treatment decisions are often based on case reports. Thus, it is critical to develop preclinical models of RMC to better understand the pathogenesis of this disease and to identify effective forms of therapy. Two novel cell line models, UOK353 and UOK360, were derived from primary RMCs that both demonstrated the characteristic SMARCB1 loss. Both cell lines overexpressed *EZH2* and other members of the polycomb repressive complex and EZH2 inhibition in RMC tumor spheroids resulted in decreased viability. High throughput drug screening of both cell lines revealed several additional candidate compounds, including bortezomib that had both in vitro and in vivo antitumor activity. The activity of bortezomib was shown to be partially dependent on increased oxidative stress as addition of the N‐acetyl cysteine antioxidant reduced the effect on cell proliferation. Combining bortezomib and cisplatin further decreased cell viability both in vitro and in vivo that single agent bortezomib treatment. The UOK353 and UOK360 cell lines represent novel preclinical models for the development of effective forms of therapy for RMC patients.

## 1 INTRODUCTION

Renal cell carcinoma (RCC) affects nearly 300 000 individuals worldwide each year with over 100 000 deaths annually. RCC consists of several distinct subtypes that vary in incidence and demonstrate associations with specific histologic, genetic, and clinical features. Renal medullary carcinoma (RMC) is a rare and aggressive subtype of RCC that predominantly afflicts individuals of African or Mediterranean decent with sickle cell trait.^1^, ^2^ Although RMC comprises less than 1% of all RCC, it is the deadliest subtype with the median overall survival of 13 months.^1^, ^3^, ^4^ This necessitates a concerted effort to develop a mechanistically‐driven approach to therapy for this cancer. Patients are most often young, with a median age from 19 to 22 years old and more frequently male with a 2:1 male to female ratio, similar to the ratios observed in other RCC subtypes.^2^, ^4^, ^5^, ^6^ RMC occurs more frequently within the right kidney for reasons that have yet to be elucidated.^2^, ^7^ While patients can present with gross hematuria or flank pain, RMC has a propensity to metastasize early, often resulting in the presentation of late stage disease with poor prognosis.^2^, ^4^, ^6^

A central feature of RMC is loss of expression of the SWI/SNF‐related matrix‐associated actin‐dependent regulator of chromatin subfamily B member 1 (SMARCB1) protein, also known as integrase interactor 1 (INI1), BRG1‐associated factor 47 (BAF47), or sucrose non‐fermenting 5 (SNF5).^8^, ^9^, ^10^ The SMARCB1 protein, encoded by *SMARCB1* in chromosome band 22q11.23, is a core subunit of the SWI/SNF chromatin remodeling complex. Loss destabilizes the complex and disrupts its equilibrium with the polycomb repressive complex 2 (PRC2) resulting in dysregulation of transcription activity.^11^, ^12^ In addition to RMC, malignant rhabdoid tumors (MRTs) also are characterized by complete inactivation of the *SMARCB1* gene.^13^ MRTs are rare and aggressive pediatric renal tumors with an estimated 80%‐90% of children succumbing to the disease, most within a year of diagnosis.^14^ It has been shown that tumors with mutation of the SWI/SNF complex subunits have a dependency on EZH2, a component of the PRC2 complex, and studies of EZH2 inhibitors in *SMARCB1* mutant MRT mouse xenograft models have shown complete responses.^15^, ^16^ Due to the low incidence of RMC and the disease's aggressive nature, large clinical trials have been difficult to perform, and treatment decisions are often based on a limited number of published case reports. A handful of reports have suggested that either bortezomib or platinum‐based combination chemotherapies, such as MVAC (methotrexate, vinblastine, doxorubicin, and cisplatin), could provide a therapeutic option.^6^, ^17^, ^18^, ^19^ Currently, there is a need for cell line models for RMC to provide a crucial tool for the investigation of novel therapies for patients affected with RMC. A recent study by Hong et al^20^ managed to produce two primary tumor cell lines from two separate patients using a ROCK inhibitor (Y‐27632) based methodology^21^ to produce a successful culture. Both cell lines and the tumors from which they were derived had the expected loss of SMARCB1 function and the cell lines demonstrated an in vitro response to bortezomib.^20^ Neither of the primary tumor lines developed in this study were capable of producing tumor xenografts and so this study relied on utilizing the SMARCB1‐deficient rhabdoid cell line G401 as a substitute for an actual RMC cell line in its in vivo studies.^20^ Confirmation of in vitro data by in vivo analysis is fundamentally important for the evaluation of therapeutic agents; thus, a model that allows for in vivo analysis is essential.

We endeavored to establish new RMC cell lines in hopes of elucidating their biology and defining effective treatments. Herein we report the development of two patient tumor‐derived cell lines, UOK353 and UOK360, that resulted from spontaneous transformation and represent models for RMC. These cell lines allow the adoption of a two‐pronged approach to identification of potential therapeutics using (1) a targeted approach focused on exploiting *SMARCB1* loss and (2) high throughput drug screening. Both cell lines produced tumor xenografts in nude mice, with UOK360 more rapidly, providing models for both in vitro and in vivo analysis of potential therapeutic targets.

## 2 MATERIALS AND METHODS

### 2.1 Patient material

Patients were evaluated and managed at the Hatfield Clinical Research Center, National Institutes of Health (NIH). Tumor tissues were obtained from biopsy or surgical resection and used for cell line production. Peripheral blood and tumor samples were obtained for DNA extraction. All patients signed the Urologic Oncology Branch protocol, NCI‐97‐C‐0147 that has been approved by the Institutional Review Board of the National Cancer Institute and that includes the production and use of cell line models. Informed consent was acquired only after the patient had fully understood what might be done with samples obtained from them and the implications of our research.

### 2.2 Cell line production

Surgically resected tumor tissue from one patient and a tumor biopsy from a second patient were utilized to establish two cell lines, UOK353 and UOK360, in accordance with protocols and techniques previously described by the Urologic Oncology Branch.^22^ Sterile tumor tissues were acquired and transferred to tissue culture as rapidly as possible to maximize the viability of the tissue. Both cell lines were propagated for over 20 passages before analysis. UOK353 was found to have a doubling time of approximately 72 hours, while UOK360 had a doubling time of approximately 24 hours. All cells were cultivated in high glucose (25 mM d‐glucose) DMEM medium supplemented with 10% fetal calf serum and 2 mM l‐glutamine. Short tandem repeat (STR)‐based DNA‐profiling was performed on the cell lines and the matched peripheral blood DNA to confirm the origins of both cell lines.

### 2.3 Spectral karyotyping (SKY) and fluorescence in situ hybridization (FISH)

FISH and SKY were performed on both UOK353 and UOK360, on interphase and metaphase spreads, respectively. Interphase cells were hybridized with a SMARCB1 break apart probe (#SMARCB1BA‐20‐GROR, Empire Genomics, Williamsville, NY). Images were imaged on ×63 objective using a Leica Thunder Imager (Leica Microsystems, Wetzlar, Germany). Metaphase spreads (n = 25) were scored for chromosomal copy number and for structural aberrations as previously described.^23^ Spectrum‐based classification and analysis of the fluorescent images was performed by using SkyView software (Applied Spectral Imaging, Carlsbad, CA). The chromosome complements of every metaphase spread were analyzed and the karyotypes were described according to human chromosome nomenclature standards described in ISCN 2009.^24^ Structural aberrations and numerical chromosomal gains were considered clonal if two or more cells contained the same change, while numerical chromosomal losses were considered clonal if three or more cells demonstrated the same loss.^23^, ^24^

### 2.4 Gene panel sequencing

DNA was extracted from cell pellets, tumors, and peripheral blood leucocytes using a Promega Maxwell 16 Cell, Tissue, or Blood DNA Purification Kit (Promega, Fitchburg, WI). The UOK353 and UOK360 cell line DNAs were assessed using the OncoVar v.4 assay provided by Genetics Branch, National Cancer Institute (NCI). The assay performs hybrid capture sequencing analysis for genomic variants on a panel of cancer‐related genes, including the known kidney cancer associated genes.^25^, ^26^ Mutations of the *SMARCB1* and *TP53* genes identified by the OncoVar v.4 assay were validated by an orthogonal sequencing methodology to validate the accuracy of the mutation calling. Sanger sequencing was performed on PCR products generated using a Qiagen Taq PCR Core Kit (Qiagen, Germantown, MD) that were bidirectionally sequenced using the BigDye Terminator v.1.1 Cycle Sequencing Kit (Thermo Fisher Scientific, Waltham, MA), in accordance with manufacturer's protocols. Sequence reactions were cleaned with Performa DTR Plates (Edge Bio, Gaithersburg, MD) and capillary electrophoresis was performed on an ABI 3730/ABI 3130xl Genetic Analyzer automated sequencing machine (Applied Biosystems). Sanger Sequencing was conducted at the CCR Genomics Core, NCI. Forward and reverse sequences were evaluated using Sequencher 5.4.6 (Genecodes, Ann Arbor, MI).

### 2.5 RNA extraction and real‐time PCR analysis

Total RNA was extracted from cell lines grown in 10 cm dishes using Direct‐zol Miniprep (Zymo Research, Irvine, CA), following a standard protocol. Cell lines were grown to a confluency of approximately 80%‐90%, washed with 5 mL of sterile PBS and lysed using 1 mL of Trizol reagent. Following the standard RNA extraction protocol, the RNA was resuspended in 50 μL of RNase‐free water and the RNA concentration was measured using a NanoDrop 2000 UV‐Vis Spectrophotometer (Thermo Fisher Scientific). For each cell line, cDNA was synthesized from 1 μg of total mRNA using the SuperScript VILO cDNA Synthesis Kit (Thermo Fisher Scientific) in a 20 μL volume. The cDNAs were diluted 10‐fold with 180 μL of RNase‐free water and 2 μL was used in 10 μL reaction volume for RT‐PCR amplification using an ABI ViiA7 real‐time PCR system (Thermo Fisher Scientific). Expression levels were normalized to the control 18S housekeeping gene (Hs99999901_s1) and calculated using the ViiA7 software as comparative CT (ΔΔCT) values. The non‐immortalized normal kidney cell line HRCE was designated to represent the normal expression level with a value of 1. TaqMan Gene Expression Assays (Thermo Fisher Scientific) were used to assess the expression levels of several genes, including components of the SWI/SNF complex—*SMARCB1* (Hs00992521_m1), *SMARCA4* (Hs00946396_m1), and *PBRM1* (Hs00217778_m1), components of the PRC2 complex—*EZH2* (Hs01016789_m1), *SUZ12* (Hs00248742_m1), and *EED* (Hs00537777_m1), and downstream expression markers of activation of the NRF2‐antioxidant response element (ARE) signaling pathway—*NQO1* (Hs00168547_m1), *HMOX1* (Hs01110250_m1), *SQSTM1 (p62)* (Hs01061917_g1), *GCLC* (Hs00155249_m1), and *GCLM* (Hs00978072_m1).

### 2.6 Western blotting

Sub‐confluent cells were trypsinized and washed once in PBS. Total proteins were extracted by solubilizing the cell pellets in urea buffer (8 M urea, 0.1 M NaH_2_PO_4_, 10 mM Tris pH 8, 0.1% mercaptoethanol). Protein concentration was quantified by the Bio‐Rad protein assay (Bio‐Rad Laboratories, Hercules, CA). Proteins (20 μg) were separated by electrophoresis on NuPage 4%‐20% Bis‐Tris gels (Life Technologies Corporation, Carlsbad, CA) and transferred onto Immobilon‐P membranes (Millipore Sigma, Burlington, MA) according to the manufacturers' directions. Western blot analyses of proteins were carried out by using anti‐Ku80 (C48E7) (Cell Signaling Technology Inc., Danvers, MA), anti‐BAF180 (ABE70) (Millipore Sigma), anti‐BRG1/SMARCA4 (SC‐17796) (Santa Cruz Biotechnology, Santa Cruz, CA), anti‐BAF57/SMARCE1 (A300‐810A) (Bethyl Laboratories, Montgomery, TX), anti‐SNF5/ SMARCB1 (612111) (BD Biosciences, San Jose, CA), anti‐EZH2 (ab3748) (Abcam, Cambridge, MA), anti‐SUZ12 (ab126577) (Abcam), and anti‐EED (ab4469) (Abcam). The primary antibodies were detected using fluorescently labeled anti‐mouse and anti‐rabbit secondary antibodies at a dilution of 1:10 000 and obtained from Li‐Cor and were visualized by scanning the blots on a Li‐Cor Odyssey (Thermo Fisher Scientific).

### 2.7 Drug treatment and growth assay protocol

The cells were seeded onto 96‐well plates and treated with the indicated drug with multiple replicates for 48 hours. Cell proliferation was measured by Cell Titer Glo (Promega) at 48 hours posttreatment. The Cell Titer Glo assay was conducted according to the manufacturer's instructions.

### 2.8 Tumor spheroid generation and drug treatment

Cell lines were evaluated using several EZH2 inhibitors, GSK126, EPZ6438, and JQEZ5, but these agents require an extended time period to be effectively evaluated that is not compatible with 2D in vitro assays in these fast‐growing cell lines, so 3D spheroid viability assays were utilized.^28^ Tumor spheroids were seeded into Ultra‐Low Attachment (ULA) 96 well plates (Corning 4530, Kennebunk, ME, Nexcelom ULA‐96U‐20, Lawrence, MA) at spheroid forming density. The cells were incubated at 37°C, 5% CO_2_ until formation was confirmed by eye under a microscope. UOK353 did not produce usably spheroids, only loose aggregates, and where not analyzed. However, UOK360 cells produced tight spheroids and these were evaluated with the positive control SMARCB1‐deficient MRT cell line G401. After spheroids had been determined to have formed, drugs were added at ×2 concentration to reach ×1. All drugs were incubated for 48 hours and evaluated using Cell Titer Glo3D. For GSK126, every 3‐4 days 100 μL of the 200 μL of media in each well was removed and replaced with 100 μL fresh media containing ×2 the desired drug concentration. Cell Titer Glo 3D assay was conducted according to the manufacturer's instructions.

### 2.9 Combination index calculation

Samples were treated with equimolar concentration of the indicated compounds and viability as determined by Cell Titer Glo was utilized as the endpoint. The combination index calculation was performed using CompuSyn software (ComboSyn, Inc., Paramus, NJ).

### 2.10 Mouse xenograft protocol

Approximately 5 × 10^6^ UOK353 or 1‐3 × 10^6^ UOK360 cells were suspended in a 0.2 mL mixture of 50% PBS and 50% Matrigel Matrix (Corning Life Sciences, Tewksbury, MA) and subcutaneously injected into 10 NCI Athymic NCr‐nu/nu mice (obtained from Charles River Frederick Research Model Facility) to evaluate the tumorigenic potential of these cell lines. All animal care protocols used had been approved by the Institutional Animal Care and Use Committee (IACUC) and were in accordance with National Cancer Institute guidelines.

### 2.11 Quantitative high throughput screening

Quantitative high throughput screening (qHTS) was conducted with the assistance of the National Center for Advancing Translational Sciences, NIH. Five hundred UOK353 or UOK360 cells were plated into white, solid‐bottom, 1536‐well tissue culture treated plates using a Multidrop Combi cassette dispenser. The cells were then treated with the compounds of the MIPE library. Viability was assessed using Cell Titer‐Glo assay (Promega) 48 hours posttreatment and luminescence was measured on a ViewLux plate reader (PerkinElmer, Waltham, MA).

### 2.12 Invasion assay

Invasion assays were performed using the xCELLigence system as previously described^27^ with the following modifications (ACEA Biosciences, Inc., San Diego, CA). Following overnight serum starvation, 35 000 UOK353 or UOK360 cells were seeded in each well of the upper chamber in media without FBS. DMEM with 10% FBS was placed in the lower chamber. Serial dilutions of medications were established as documented. The impedance value of each well was continually monitored every 15 minutes by the xCELLigence system for 96 hours following seeding.

### 2.13 CellROX® green assay

CellROX® green reagent is a cell‐permeant dye used for measuring oxidative stress in live cells (Thermo Fisher Scientific Inc.). The probe is weakly fluorescent while in a reduced state and exhibits bright green photostable fluorescence upon oxidation by ROS. Cells were evaluated at 48 hours posttreatment.

## 3 RESULTS

### 3.1 Development of the UOK353 and UOK360 renal medullary carcinoma cell lines

**FIGURE 1:**
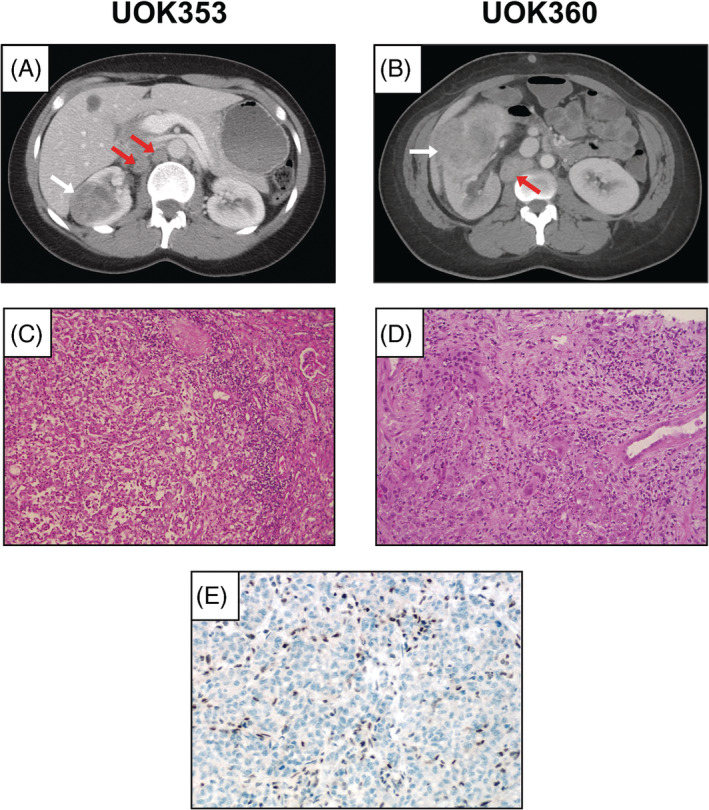
Clinical imaging and histopathology of renal medullary carcinoma patients. A and B, Axial CT image of a right renal mass (white arrow) and two lymph nodes with metastatic disease (red arrows) in the patient from which UOK353 was derived. H&E staining of the resected renal mass was consistent with renal medullary carcinoma. C, Immunohistochemical staining for INI1 (SMARCB1) demonstrated no signal from the tumor cells in the right renal mass with positive staining seen in the infiltrating normal cells. D and E, Axial CT image of a large right renal mass (white arrow) and metastatic lymph node (red arrow) in the patient from which UOK360 was derived. H&E staining of the sample gained by needle biopsy of the renal mass was consistent with renal medullary carcinoma

UOK353 was derived from a 42‐year‐old female, African‐American patient with sickle‐cell trait who initially presented with flank pain and was found to have a 4.1 cm right kidney mass and bulky retroperitoneal lymphadenopathy (Figure 1A). The patient underwent a radical nephrectomy removing a 5.1 cm mid/lower pole pT3a tumor, as well as six para‐aortic and two interaortocaval lymph nodes with metastatic renal cell carcinoma. Histopathology staining of the primary tumor was consistent with RMC and had positive immunohistochemistry staining for CEA and negative staining for CK7 and INI‐1 (SNF5/SMARCB1) (Figure 1B,C). UOK360 was derived from a 41‐year‐old female, African American patient with sickle‐cell trait who presented with anemia and weight loss. Evaluation revealed an 8 cm solid mass in the right kidney, bulky retroperitoneal lymphadenopathy, and multiple pulmonary and osseous lesions suggestive of metastases (Figure 1D). A needle biopsy of the right kidney mass demonstrated histopathology consistent with RMC (Figure 1E), with positive immunohistochemistry staining for CEA and negative staining for CK7 and INI‐1 (SNF5/SMARCB1).

Procured tissue from the surgery‐resected primary renal tumor of the first patient and biopsy tissue from the second patient was used to derive two new, spontaneously immortal cell lines (grown for more than 20 passages), UOK353 and UOK360 respectively.

### 3.2 Loss of SMARCB1/SNF5 expression in UOK353 and UOK360

**FIGURE 2:**
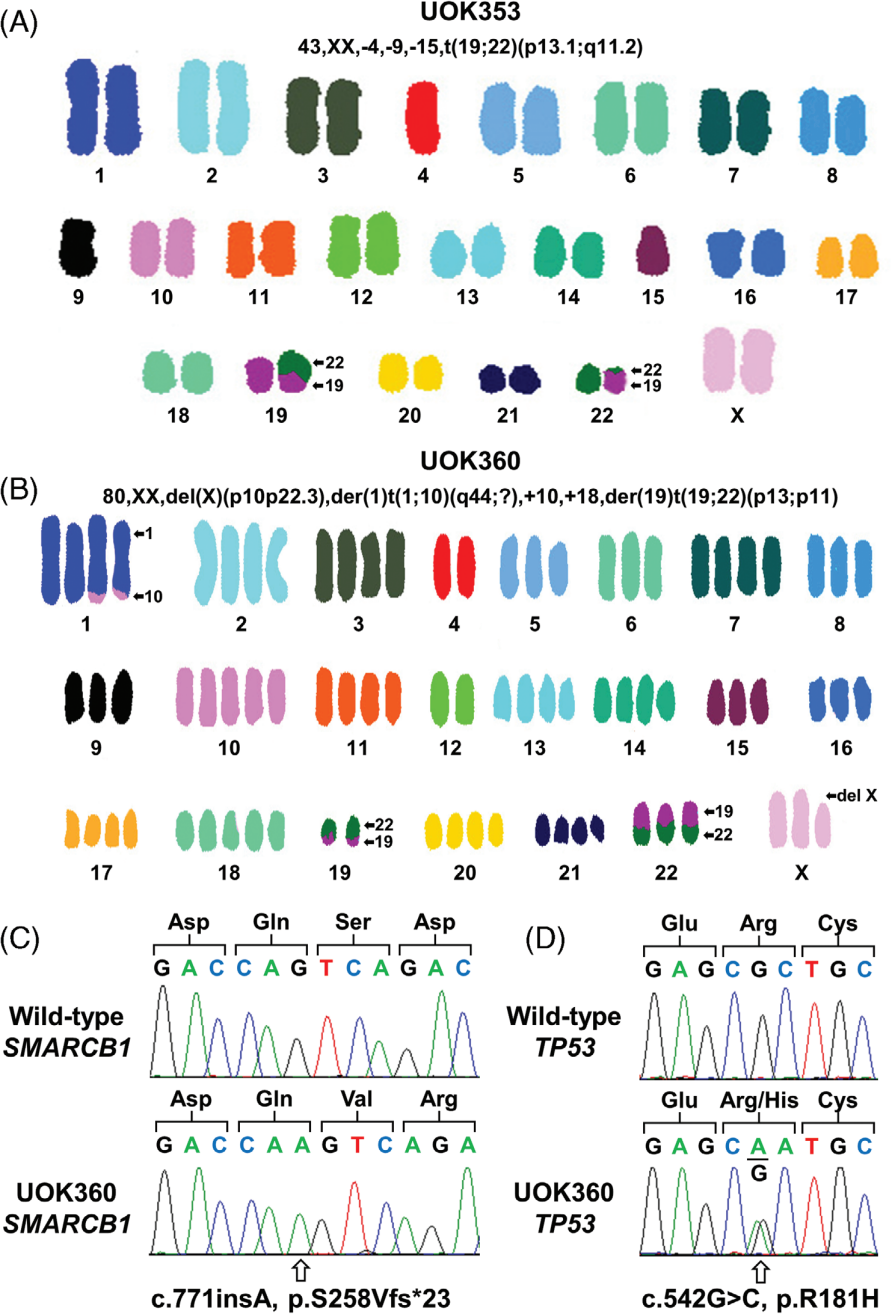
Karyotype and mutation analysis of UOK353 and UOK360. A and B, Spectral karyotype (SKY) of both UOK353 and UOK360 demonstrated chromosomal level alterations, including balanced translocations between chromosomes 19 and 22 in both cell lines. In UOK353, the translocation occurred in the 22q11.2 sub‐band that contains the *SMARCB1* gene. C, A somatic homozygous *SMARCB1* frameshift mutation was observed in UOK360. D, A somatic heterozygous *TP53* missense mutation in a known pathogenic hotspot was observed in UOK360

Loss of INI‐1 (SMARCB1/SNF5) immunohistochemistry staining was observed in both patient tumors. The mechanisms of this loss were investigated with spectral karyotypic (SKY) and genomic analysis. SKY analysis of UOK353 revealed that the modal number of chromosomes was hypodiploid, with consistent loss of one copy of chromosomes 4, 9, and 15 (Figure 2A). Translocation between chromosomes 19 and 22, t(19;22)(p13.1;q11.2), produced a chromosomal break in the region of the *SMARCB1* gene (22q11.23) and FISH analysis using SMARCB1 break apart probes demonstrated that the break occurred within the *SMARCB1* gene resulting in loss of activity (Figure S1). The FISH analysis did not show any wild‐type signal in UOK353 and TaqMan‐based copy number analysis demonstrated loss of one copy of *SMARCB1* that presumably represents a specific, focal deletion of the wild‐type *SMARCB1*. UOK353 mutation analysis revealed no point mutations in *SMARCB1* nor any other gene known to be associated with RCC. No promoter hypermethylation of *SMARCB1* was identified.

UOK360 SKY analysis revealed the modal number of chromosomes to be hypertriploid to near‐tetraploid for almost every cell, indicating a gross genome duplication event, although there was no amplification of chromosomes 4 and 12 (Figure 2B). Two consistent translocations were identified between chromosomes 1 and 10, t(1;10)(q44;?), and chromosomes 19 and 22, t(19;22)(p13;p11), in combination with the consistent deletion of the p arm of chromosome X. Although the translocation of chromosomes 19 and 22 occurred at a distance from *SMARCB1* (Figure S1), only the derivative chromosomes were present due to loss of the wild‐type chromosomes 19 and 22. Mutation analysis of UOK360 demonstrated a homozygous adenine insertion (c.771insA) mutation of *SMARCB1*, resulting in a frameshift that produced a truncated protein (p.S258Vfs*23) (Figure 2C). In addition, UOK360 was revealed to have a heterozygous missense (c.542G>C) mutation in *TP53*, resulting in an altered protein (p.R181H) at a known cancer mutation hotspot (Figure 2D).

### 3.3 Upregulation of the PRC2 complex and response to EZH2 inhibitors

**FIGURE 3:**
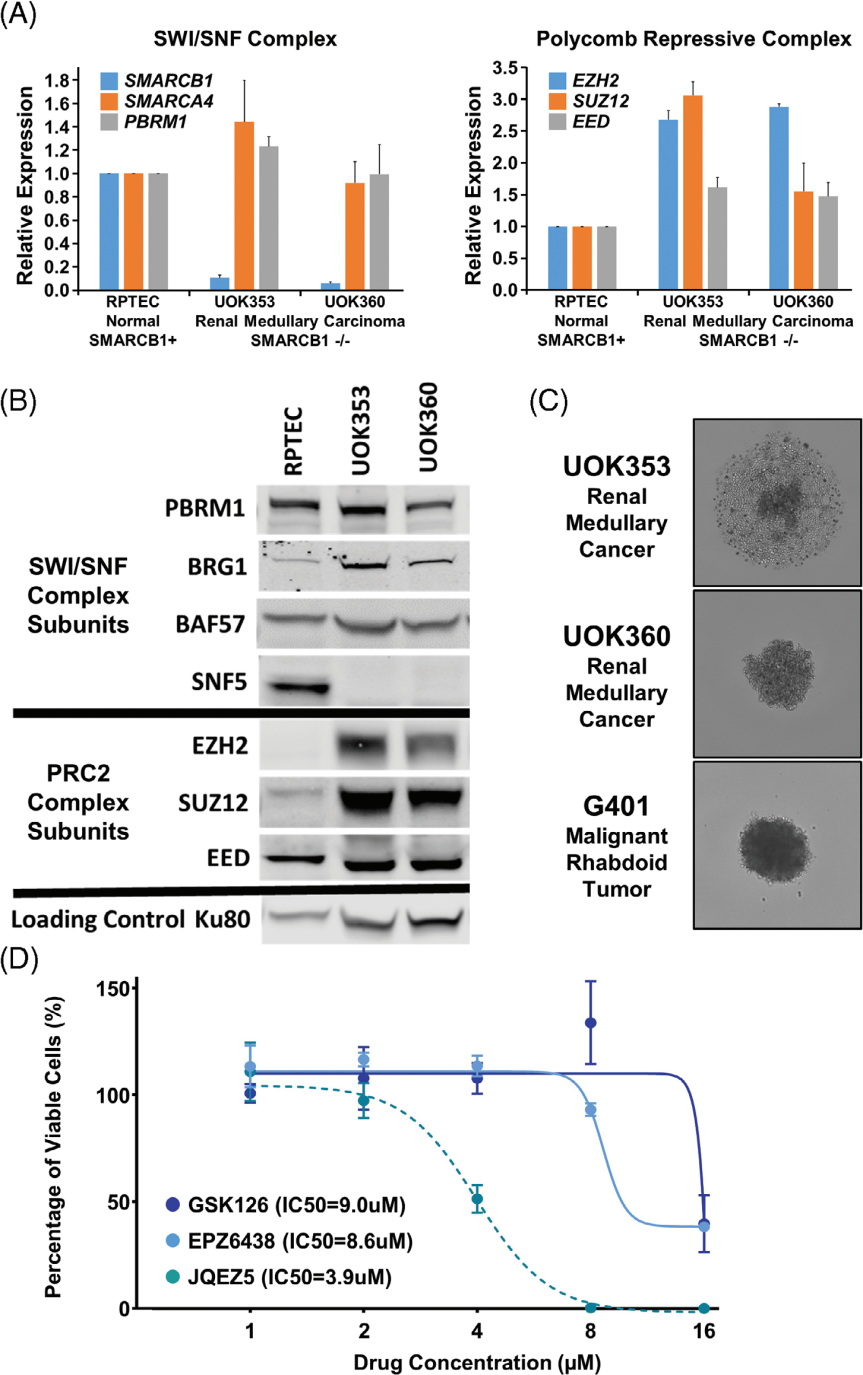
Evaluation of EZH2 inhibition in renal medullary carcinoma cell lines. A, Real‐time PCR analysis of the UOK353 and UOK360 cell lines demonstrated loss of *SMARCB1* expression relative to the normal control cell line RPTEC (renal proximal tubule epithelial cells), while expression levels of other SWI/SNF components were normal. In contrast, multiple PRC2 subunits (*EZH2*, *SUZ12*, *EED*) were overexpressed relative to RPTEC. B, These changes were confirmed at the protein level as determined by immunoblotting. C, The RMC cell lines were assessed for tumor spheroid forming ability by culturing in 96‐well ultra‐low attachment plates. UOK360 cells formed tight spheroids, similar to the malignant rhabdoid tumor cell line G401 that was used as a reference. UOK353 cells only formed a loose aggregate on a lawn of unincorporated cells and were not considered suitable for 3D viability analysis. D, IC50 curves were calculated for UOK360 tumor spheroids over a period of 14 days in response to treatment with three published, commercially available EZH2 inhibitors, GSK126, EPZ6438, and JQEZ5

Previous studies in malignant rhabdoid tumor (MRT) have shown that the loss of SWI/SNF complex subunits, such as SMARCB1/SNF5, result in an up‐regulation and dependency upon an opposing chromatin remodeling complex, the polycomb repressive complex 2 (PRC2).^13^, ^14^, ^15^, ^16^ Both cell lines exhibited extremely low levels of *SMARCB1* gene mRNA expression, relative to primary renal proximal tubule epithelial cells (RPTEC), and no detectable levels of SMARCB1/SNF5 protein by western blot analysis (Figure 3A,B). However, the mRNA and protein expression for other SWI/SNF subunits, such as PBRM1 and SMARCA4/BRG1, were retained (Figure 3A,B). Conversely, the expression of several PRC2 subunits, EZH2, SUZ12, and EED, were upregulated at both the mRNA and protein levels in both cell lines in comparison to RPTEC cells (Figure 3A,B). Due to the similar loss of *SMARCB1* and upregulation of EZH2 shared between MRTs and RMCs, the EZH2 inhibitors GSK126, EPZ6438, and JQEZ5 were investigated as potential targeted therapies. EZH2 inhibition requires an extended time period to be effectively evaluated,^28^ and as such was not compatible with 2D in vitro assays in these fast‐growing cell lines, so 3D spheroid viability assays were utilized. UOK353 only produced loose aggregates; however, UOK360 cells produced tight spheroids and these were evaluated with a positive control SMARCB1‐deficient MRT cell line, G401 (Figure 3C). After 14 days, the UOK360 spheroids exhibited a dose‐dependent response to JQEZ5 with an IC50 of 3.9 μM, while responses to GSK126 and EPZ‐6438 were only observed at higher doses, IC50s of 9.0 and 8.6 μM, respectively (Figure 3D). While the GSK126 EZH2 inhibitor had a limited effect on the UOK360 cells, it significantly reduced viability of the G401 cells (<10% of untreated cells) at all drug concentrations suggesting variability in response to EZH2 inhibitor between MRT and RMC cells that highlights the importance of evaluating representative cell line models (Figure S2).

### 3.4 High‐throughput drug screening of UOK353 and UOK360

**FIGURE 4:**
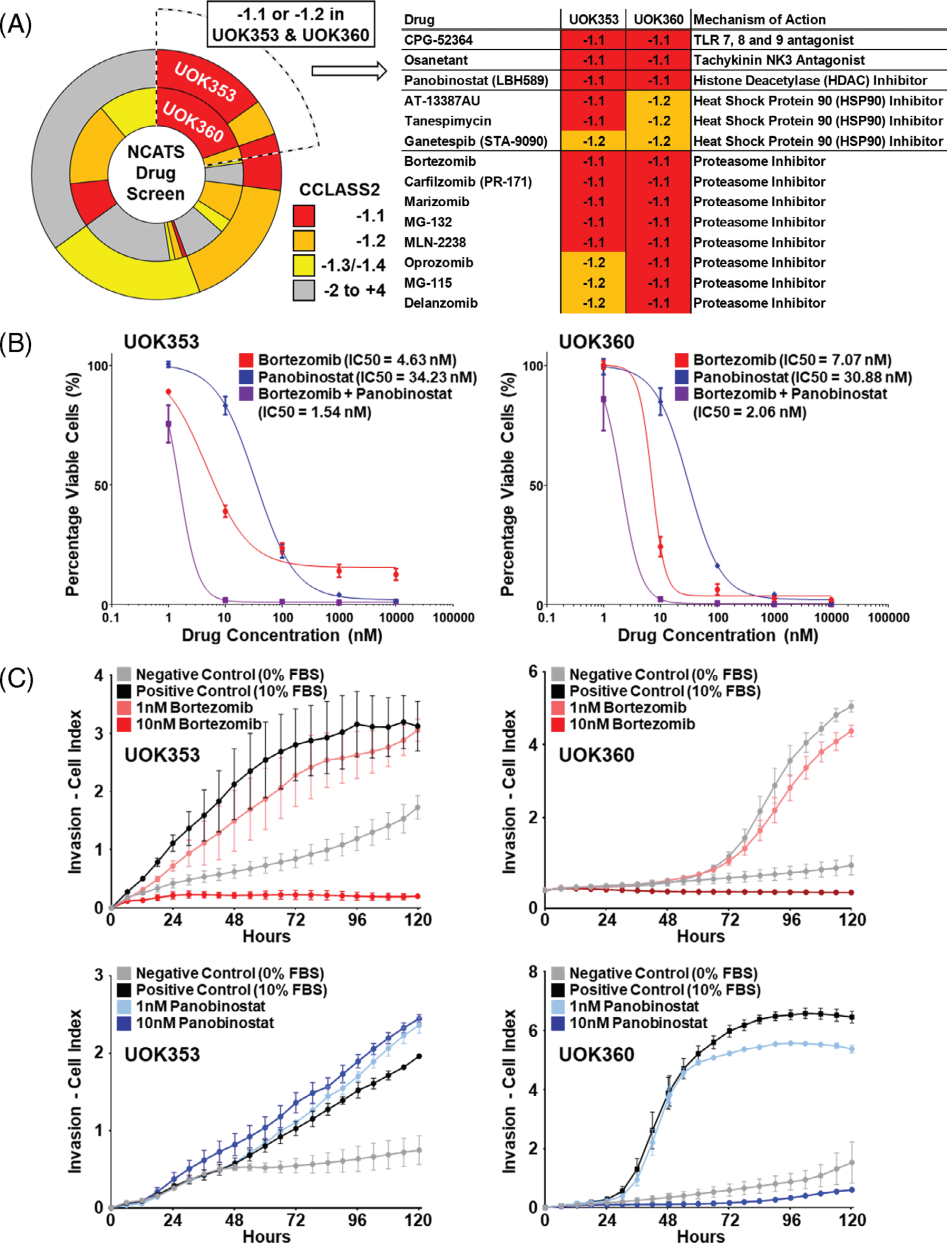
High throughput drug screening. A, High throughput screening identified several classes of candidate compounds based on the slope of their corresponding IC50 curve with CCLASS2 scores of −1.1 or −1.2 considered significant. The table highlights the compounds with significant IC50s in both cell lines. B, Confirmation assays demonstrated low IC50 concentrations for bortezomib and panobinostat in both cell lines and that the combination of bortezomib and panobinostat at equal concentrations lowered the IC50 in comparison to either single agent alone. C, Invasion assays using the xCELLigence system demonstrated that 10 nM of bortezomib inhibited invasion of both UOK353 and UOK360 but 10 nM of panobinostat only inhibited invasion of UOK360 and not UOK353. Doses of 1 nM of either agent had little effect on invasion in either cell line. Negative control wells containing media with no FBS demonstrated limited invasion in either cell line

In collaboration with the National Center for Advancing Translational Science (NCATS), a high throughput drug screen utilizing a library of FDA approved drugs and compounds currently undergoing clinical trials was performed on UOK353 and UOK360 cells in 2D culture. These screens identified several candidate drugs that were effective in both cell lines, including the toll‐like receptor 7/8/9 antagonist CPG‐52364, the neurokinin 3 receptor antagonist osanetant, and the histone deacetylase (HDAC) inhibitor panobinostat that were selected for confirmation (Figure 4A). Furthermore, two classes of compounds, heat shock protein 90 (HSP90) inhibitors and proteasome inhibitors, had consistent effects across both cell lines with AT‐13387AU and bortezomib respectively selected as examples for each class (Figure 4A). Two‐D culture demonstrated that only bortezomib and panobinostat were highly effective in reducing cell viability at low concentrations, with IC50s of 4.63 and 34.23 nM, respectively, in UOK353 and 7.07 and 30.88 nM, respectively, in UOK360 (Figures 4B and S3A). The combination of bortezomib and panobinostat in the 2D cultures produced a synergistic effect in both cell lines with combination indices of <1 by CompuSyn software analysis (Figure 4B). Cell invasion assays showed inhibition of invasion by bortezomib in a dose‐dependent manner in both RMC cell lines; however, panobinostat only inhibited the invasion of UOK360 (Figure 4C). A combination of bortezomib and panobinostat in UOK360 further inhibited invasion at lower doses in comparison to the response to a single agent, but little additional effect was observed at higher doses (Figure S3B). This suggested bortezomib as a better candidate for further studies. This is consistent with reports of bortezomib's clinical use in patients with RMC and the recent report of bortezomib's effect on primary RMC cell lines.^17^, ^18^, ^21^, ^29^

### 3.5 Evaluation and modification of the response to bortezomib

**FIGURE 5:**
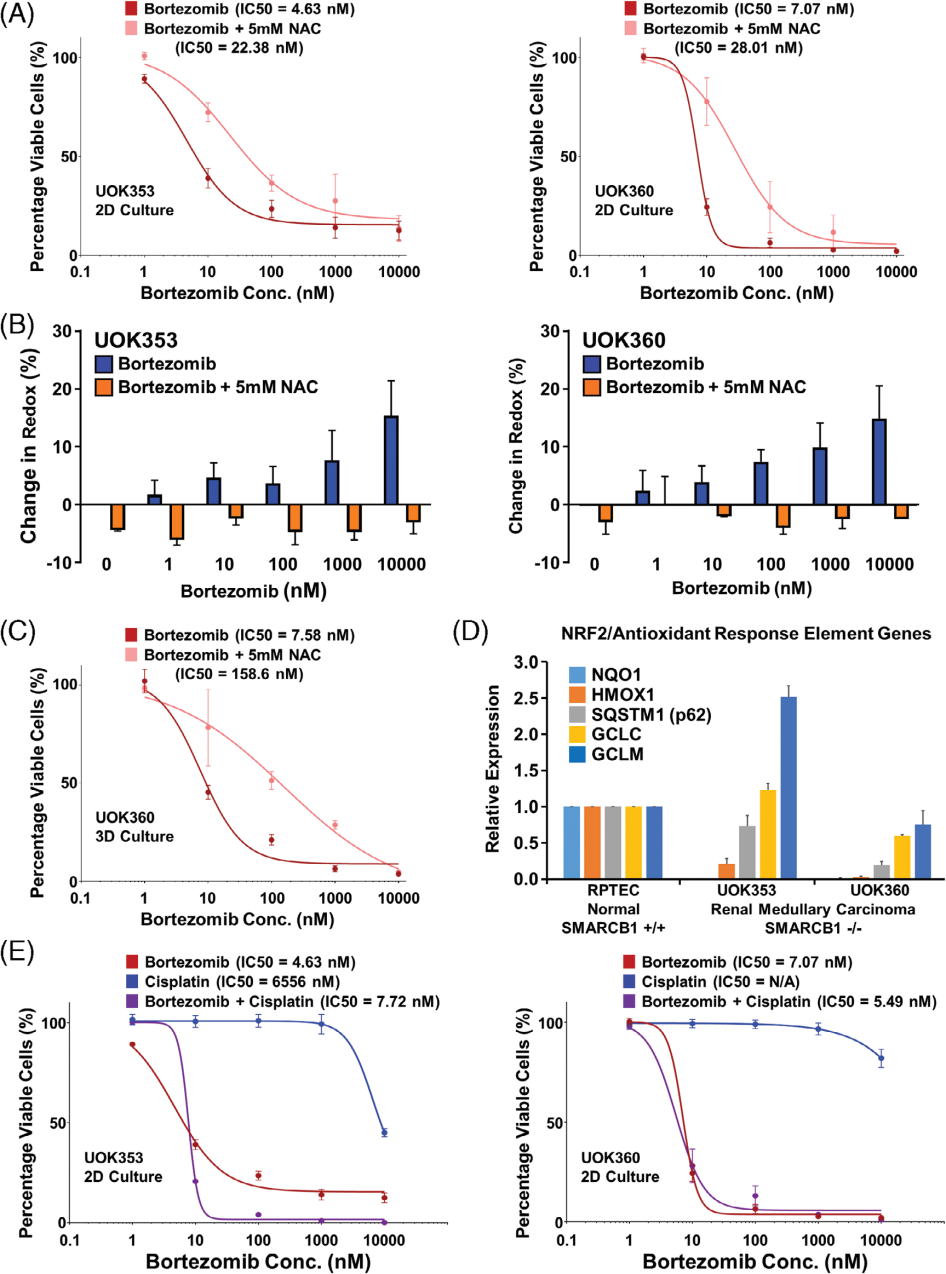
Influences on the effects of bortezomib on renal medullary carcinoma cell lines. A, Combining bortezomib with a standard dose of 5 mM of a ROS reducing agent, the antioxidant N‐acetyl‐cysteine (NAC), increased the viability of cells treated with a range of concentrations of bortezomib. B, CellRox® assays measured at 48 h posttreatment demonstrated a dose‐dependent increase in redox in response to bortezomib that was abrogated by the addition of 5 mM NAC at all concentrations of bortezomib in both UOK353 and UOK360. C, Combining bortezomib with 5 mM of NAC in the 3D culture of UOK360 spheroids increased the IC50. D, Real‐time PCR analysis of genes involved in the NRF2/antioxidant in the UOK353 and UOK360 cell lines in comparison the RPTEC normal kidney control cell line. E, Cisplatin as a single agent was shown to have little effect on either UOK353 or UOK360, but equimolar concentrations of bortezomib and cisplatin had a greater effect than bortezomib alone in UOK353 and lowered the IC50 in UOK360

Bortezomib‐associated antitumor effect has been previously correlated with increased levels of cellular ROS and improved efficacy in combination with platinum‐based therapies, such as cisplatin.^27^ The role of cellular ROS in response to bortezomib treatment was evaluated by the addition of a ROS reducing agent, the antioxidant N‐acetyl‐cysteine (NAC). The addition of 5 mM NAC was partially protective to the effects of bortezomib in 2D culture for both UOK353 and UOK360, raising the IC50s from 4.63 to 22.38 nM and 7.07 to 28.01 nM, respectively (Figure 5A). CellROX® Green analysis of ROS levels demonstrated a dose dependent increase in ROS in response to bortezomib that was completely abrogated by the addition of 5 mM NAC in all treatment concentrations (Figure 5B). The same response was observed in the 3D culture of UOK360 raising the IC50 for bortezomib from 7.58 to 158.6 nM with the addition of 5 mM NAC (Figure 5C). This suggests that bortezomib acts in a multifactorial manner that includes increased ROS generation in addition to other mechanism(s) that have not yet been identified. A known mechanism for regulating ROS levels in cells is the NRF2‐ARE signaling pathway that controls expression of several genes, including *NQO1*, *HMOX1*, *SQSTM1*, *GCLC*, and *GCLM*. Notably, both cell lines demonstrated extremely low basal expression levels for *NQO1* and reduced expression of *HMOX1*, while other members of the NRF2‐ARE signaling pathway demonstrated expression comparable to RPTEC cells (Figure 5D). The low expression of these genes may sensitize these cell lines to the increased ROS induced by bortezomib and the selectivity of these losses could be associated with SWI/SNF dysregulation rather than down regulation of the entire NRF2‐ARE signaling pathway. The potential for platinum‐based therapies to improve the effectiveness of bortezomib was investigated using cisplatin. As a single agent therapy cisplatin demonstrated little effect on either RMC cell lines, but a combination of bortezomib and cisplatin at equimolar concentration yielded a combination index (CI) of <1 in both UOK353 and UOK360, suggesting synergy (Figure 5E).^30^ In the UOK353 cells the combination increased the degree of cell death achievable and in UOK360 lowered the IC50 to 5.49 nM compared to 7.07 nM with bortezomib alone. Cisplatin was also able to increase ROS as a single agent and the equimolar combination of bortezomib and cisplatin increased the ROS levels more than the same concentration of bortezomib alone (Figure S4).

### 3.6 In vivo analysis of bortezomib and cisplatin on UOK360 xenograft growth

**FIGURE 6:**
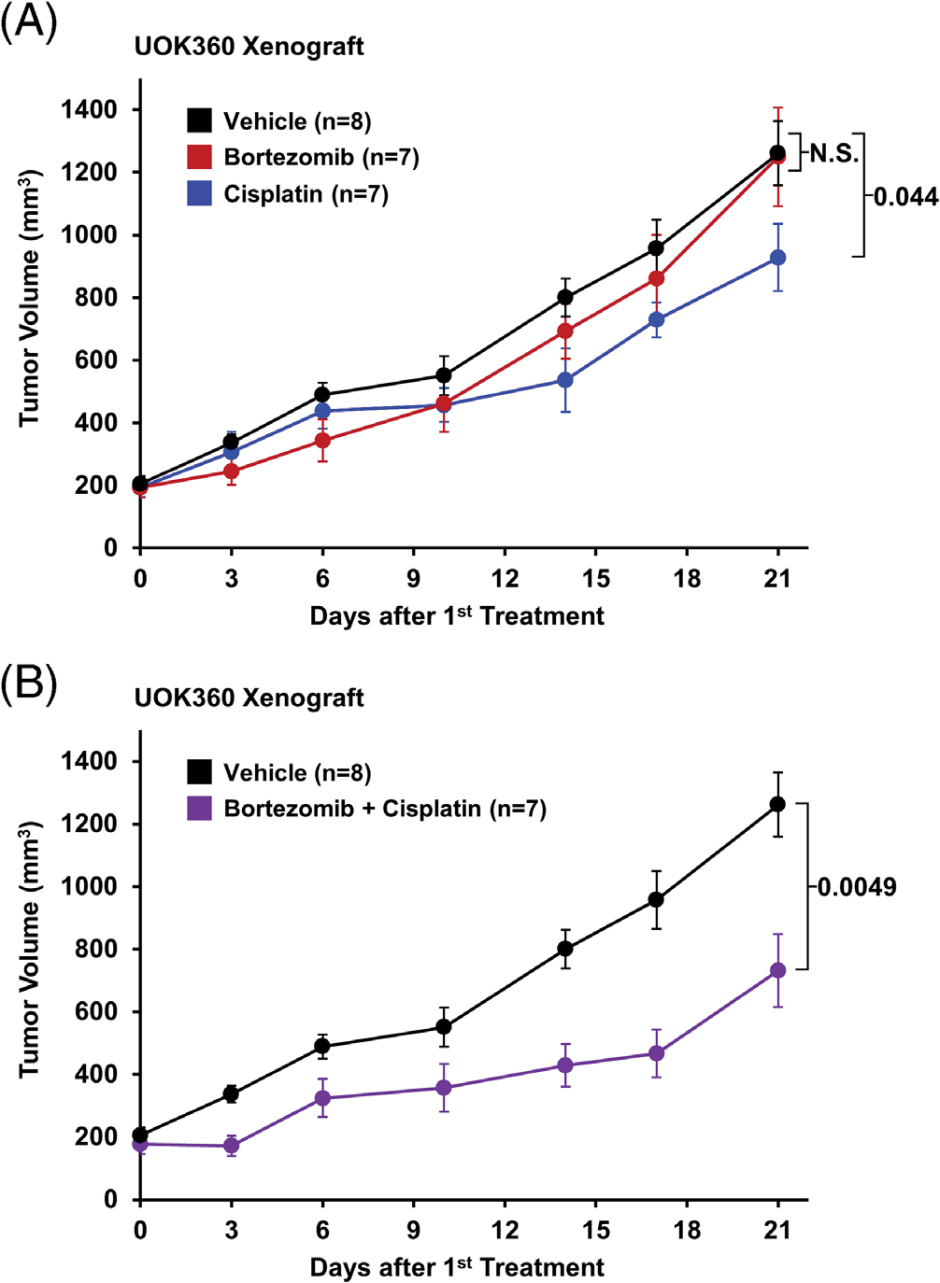
In vivo analysis of UOK360 xenografts. A and B, Mice with UOK360 xenograft tumors measuring less than 1 cm were treated by intraperitoneal (IP) injection twice weekly with either 0.9% saline vehicle (n = 8), 1 mg/kg bortezomib (n = 7), 5 mg/kg cisplatin (n = 7), or a combination of bortezomib and cisplatin (n = 7) and followed for 21 days. The size of the treated tumors after 21 days was compared to the size of the vehicle tumors by *t* test

Both UOK353 and UOK360 would produce xenograft tumors in nude mice, but UOK360 xenograft tumors grew more rapidly and produced tumors in 100% of mice. An injection of approximately 3 million UOK360 cells into the flank of athymic nude mice rapidly produced 2‐cm‐diameter xenograft tumors within 28 days. In our initial study, mice with UOK360 xenograft tumors measuring less than 1 cm were treated by intraperitoneal (IP) injection twice weekly with either 0.9% saline vehicle (n = 8), 1 mg/kg bortezomib (n = 7), 5 mg/kg cisplatin (n = 7), or a combination of bortezomib and cisplatin (n = 7) and followed for 21 days. Single agent treatment with bortezomib demonstrated little effect and with cisplatin slowed the rate of xenograft proliferation that reached statistical significance (*P* = .044) when the tumor sizes were compared to vehicle after 21 days of treatment (Figure 6A). The combination of bortezomib and cisplatin significantly reduced the size of the xenograft tumors in comparison to vehicle (*P* = .0049) with an improved suppression of the tumor growth rate compared to single agent treatment, but tumors were not eliminated (Figure 6B). A repeat study for the combination was performed using a larger number of mice (n = 13) injected with fewer cells (~1 million) and treated by IP with either 1 mg/kg bortezomib and 5 mg/kg cisplatin or 0.9% saline twice weekly for 6 weeks beginning 1 week after initial injection. This demonstrated the same trend that the combination reduced the growth rate of UOK360 xenografts but did not eliminate the tumors (Figure S5).

## 4 DISCUSSION

This study describes two novel cell line models for RMC, UOK353 and UOK360, that provide an essential tool for further elucidating the biology of RMC and providing the foundation for the development of precision therapies to target RMC. The lines were obtained from individuals who presented with the classic features of RMC with both patients being young (<45 years old), having sickle cell trait, and presenting with metastatic disease disseminating from RMC tumors that demonstrated loss of SMARCB1 (SNF5/INI‐1) staining.^1^, ^2^, ^3^, ^4^, ^5^, ^6^, ^8^, ^9^, ^10^ The loss of SMARCB1 (SNF5/INI‐1) in the UOK353 cell line correlated with a translocation between chromosomes 19 and 22 consistent with a recent study describing balanced translocations involving the *SMARCB1* locus on chromosome 22.^9^ The more aggressive, faster growing UOK360 cell line demonstrated a homozygous truncating mutation of *SMARCB1*, in conjunction with heterozygous mutation of *TP53* and aneuploidy. Although both cell lines provide models for 2D in vitro studies, the UOK360 cell line could be used for 3D in vitro study as well as rapidly producing xenograft tumors in nude mice enabling in vivo preclinical studies to be performed.

These lines therefore provide the opportunity to identify new potential treatments for RMC by investigating potential targeted therapies or utilizing high throughput drug screens. Previous studies of malignant rhabdoid tumors (MRTs) with SMARCB1/SNF5 loss had shown that those tumors upregulate expression the PRC2 complex, containing the essential subunit EZH2, and develop a significant sensitivity EZH2 inhibitors such as EPZ‐6438 (tazemetostat).^14^, ^15^, ^16^ Both RMC cell lines demonstrated similar increases in expression of PRC2 complex subunits including EZH2 but demonstrated a variable response to treatment with single agent EZH2 inhibitors. The RMC cell lines were sensitive to JQEZ5, but relatively insensitive to GSK126 and EPZ‐6438, and did not show the extreme sensitivity present in the MRT cell line model. This suggests that even though the PRC2 complex is upregulated in RMC, it may not represent the sole critical pathway for targeted therapy. Currently, there are ongoing clinical trials to evaluate tazemetostat in patients with SMARCB1‐deficient tumors including RMC and it will be very informative to compare the results of these trials with the response in the cell line models (ClinicalTrials.gov Identifiers: NCT02601937 and NCT02601950).

While the efficacy of EZH2 inhibitors in RMC is still unknown, several small‐scale studies have demonstrated a response with bortezomib or platinum‐based combination chemotherapies, such as MVAC (methotrexate, vinblastine, doxorubicin, and cisplatin).^6^, ^17^, ^18^, ^19^ Bortezomib is a known anticancer medication that is currently used to treat multiple myeloma and mantle cell lymphoma and targets the ubiquitin‐proteasome pathway (UPP) by binding the catalytic site of the 26S proteasome. Bortezomib's effect on ROS is due to the inhibition of the proteasome. The potential effectiveness of bortezomib, and other proteasome inhibitors, was identified in a high throughput drug screen performed on UOK353 and UOK360. Both cell line models showed significant responses to bortezomib and cisplatin as single agents, and in combination, these agents demonstrated a synergistic effect. In UOK360, the combination of bortezomib and cisplatin significantly reduced proliferation in both 3D culture and mouse xenografts. This response is consistent with previous reports in patients, suggesting that cell line models have the potential to predict response. Analysis of the cell lines demonstrated that the response to bortezomib was partially dependent upon the induction of increased reactive oxygen species (ROS) and cellular stress. Therefore, RMC tumors may be sensitive to other ROS inducing agents such as vandetanib, which was effective in HLRCC‐associated kidney cancer cell line models.^27^ NQO1 functions as a negative regulator of cellular ROS levels and loss of NQO1 expression was seen in both cell lines, suggesting a common mechanism for down regulation in RMC and a potential cause for sensitivity to ROS‐induction. Although the frequency of NQO1 loss in RMC tumors is currently unknown, it could provide a biomarker for specific sensitivity to treatment with ROS inducing agents.

This study also identified a response in these cell line models to panobinostat, an HDAC inhibitor, that could affect the gene expression dysregulation resulting from loss of SMARCB1/SNF5. Panobinostat inhibited proliferation and may represent a potential component of a combination therapy rather than a single agent treatment but highlights the potential for using preclinical models for drug discovery.

In summary, UOK353 and UOK360 represent two novel human cell line models for RMC and the first in vivo mouse xenograft models of RMC. These models will provide an invaluable tool for research and preclinical drug testing. Initial analysis of these models confirmed the potential for combination therapy of bortezomib and cisplatin in RMC and highlighted other potential therapeutic options for patients with advanced renal medullary carcinoma.

## CONFLICT OF INTEREST

The authors declare no conflicts of interest.

## Supporting information


**Data S1**: Supporting Information

## Data Availability

The data that supports the findings of this study are available in the supplementary material of this article or available from the corresponding author upon request.

## References

[1] Davis CJ Jr , Mostofi FK , Sesterhenn IA . Renal medullary carcinoma. The seventh sickle cell nephropathy. Am J Surg Pathol. 1995;19(1):1‐11. 7528470 10.1097/00000478-199501000-00001

[2] Hakimi AA , Koi PT , Milhoua PM , et al. Renal medullary carcinoma: the Bronx experience. Urology. 2007;70(5):878‐882. 18068443 10.1016/j.urology.2007.06.1124

[3] Maroja Silvino MC , Venchiarutti Moniz CM , Munhoz Piotto GH , Siqueira S , Galapo Kann A , Dzik C . Renal medullary carcinoma response to chemotherapy: a referral center experience in Brazil. Rare Tumors. 2013;5(3):e44. 24179656 10.4081/rt.2013.e44 PMC3804819

[4] Ezekian B , Englum B , Gilmore BF , et al. Renal medullary carcinoma: a national analysis of 159 patients. Pediatr Blood Cancer. 2017;64(11):e26609. 10.1002/pbc.26609 28485059

[5] Marsh A , Golden C , Hoppe C , Quirolo K , Vichinsky E . Renal medullary carcinoma in an adolescent with sickle cell anemia. Pediatr Blood Cancer. 2014;61(3):567. 24108577 10.1002/pbc.24795

[6] Iacovelli R , Modica D , Palazzo A , Trenta P , Piesco G , Cortesi E . Clinical outcome and prognostic factors in renal medullary carcinoma: a pooled analysis from 18 years of medical literature. Can Urol Assoc J. 2015;9(3–4):E172‐E177. 26085875 10.5489/cuaj.2373 PMC4455635

[7] Berman LB . Sickle cell nephropathy. Jama. 1974;228(10):1279. 4406521

[8] Liu Q , Galli S , Srinivasan R , Linehan WM , Tsokos M , Merino MJ . Renal medullary carcinoma: molecular, immunohistochemistry, and morphologic correlation. Am J Surg Pathol. 2013;37(3):368‐374. 23348212 10.1097/PAS.0b013e3182770406 PMC7604824

[9] Calderaro J , Masliah‐Planchon J , Richer W , et al. Balanced translocations disrupting SMARCB1 are hallmark recurrent genetic alterations in renal medullary carcinomas. Eur Urol. 2016;69(6):1055‐1061. 26433572 10.1016/j.eururo.2015.09.027

[10] Cheng JX , Tretiakova M , Gong C , Mandal S , Krausz T , Taxy JB . Renal medullary carcinoma: rhabdoid features and the absence of INI1 expression as markers of aggressive behavior. Mod Pathol. 2008;21(6):647‐652. 18327209 10.1038/modpathol.2008.44

[11] Masliah‐Planchon J , Bieche I , Guinebretiere JM , Bourdeaut F , Delattre O . SWI/SNF chromatin remodeling and human malignancies. Annu Rev Pathol. 2015;10:145‐171. 25387058 10.1146/annurev-pathol-012414-040445

[12] Kadoch C , Copeland RA , Keilhack H . PRC2 and SWI/SNF chromatin remodeling complexes in health and disease. Biochemistry. 2016;55(11):1600‐1614. 26836503 10.1021/acs.biochem.5b01191

[13] Versteege I , Sevenet N , Lange J , et al. Truncating mutations of hSNF5/INI1 in aggressive paediatric cancer. Nature. 1998;394(6689):203‐206. 9671307 10.1038/28212

[14] Roberts CW , Orkin SH . The SWI/SNF complex – chromatin and cancer. Nat Rev Cancer. 2004;4(2):133‐142. 14964309 10.1038/nrc1273

[15] Knutson SK , Warholic NM , Wigle TJ , et al. Durable tumor regression in genetically altered malignant rhabdoid tumors by inhibition of methyltransferase EZH2. Proc Natl Acad Sci U S A. 2013;110(19):7922‐7927. 23620515 10.1073/pnas.1303800110 PMC3651445

[16] Kim KH , Kim W , Howard TP , et al. SWI/SNF‐mutant cancers depend on catalytic and non‐catalytic activity of EZH2. Nat Med. 2015;21(12):1491‐1496. 26552009 10.1038/nm.3968 PMC4886303

[17] Ronnen EA , Kondagunta GV , Motzer RJ . Medullary renal cell carcinoma and response to therapy with bortezomib. J Clin Oncol. 2006;24(9):e14. 16549825 10.1200/JCO.2005.05.0344

[18] Shetty A , Matrana MR . Renal medullary carcinoma: a case report and brief review of the literature. Ochsner J. 2014;14(2):270‐275. 24940141 PMC4052598

[19] Rathmell WK , Monk JP . High‐dose‐intensity MVAC for advanced renal medullary carcinoma: report of three cases and literature review. Urology. 2008;72(3):659‐663. 18649931 10.1016/j.urology.2008.05.009

[20] Hong AL , Tseng YY , Wala JA , et al. Renal medullary carcinomas depend upon SMARCB1 loss and are sensitive to proteasome inhibition. Elife. 2019;8:e44161. 10.7554/eLife.44161 PMC6436895 30860482

[21] Liu X , Ory V , Chapman S , et al. ROCK inhibitor and feeder cells induce the conditional reprogramming of epithelial cells. Am J Pathol. 2012;180(2):599‐607. 22189618 10.1016/j.ajpath.2011.10.036 PMC3349876

[22] Yang Y , Padilla‐Nash HM , Vira MA , et al. The UOK 257 cell line: a novel model for studies of the human Birt‐Hogg‐Dube gene pathway. Cancer Genet Cytogenet. 2008;180(2):100‐109. 18206534 10.1016/j.cancergencyto.2007.10.010 PMC2440670

[23] Padilla‐Nash HM , Barenboim‐Stapleton L , Difilippantonio MJ , Ried T . Spectral karyotyping analysis of human and mouse chromosomes. Nat Protoc. 2006;1(6):3129‐3142. 17406576 10.1038/nprot.2006.358 PMC4772431

[24] Simons A , Shaffer LG , Hastings RJ . Cytogenetic nomenclature: changes in the ISCN 2013 compared to the 2009 edition. Cytogenet Genome Res. 2013;141(1):1‐6. 23817294 10.1159/000353118

[25] Sorber R , Teper Y , Abisoye‐Ogunniyan A , et al. Whole genome sequencing of newly established pancreatic cancer lines identifies novel somatic mutation (c.2587G>a) in axon guidance receptor plexin A1 as enhancer of proliferation and invasion. PLoS One. 2016;11(3):e0149833. 26962861 10.1371/journal.pone.0149833 PMC4786220

[26] Killian JK , Miettinen M , Walker RL , et al. Recurrent epimutation of SDHC in gastrointestinal stromal tumors. Sci Transl Med. 2014;6(268):268ra177. 10.1126/scitranslmed.3009961 PMC7670881 25540324

[27] Sourbier C , Ricketts CJ , Matsumoto S , et al. Targeting ABL1‐mediated oxidative stress adaptation in fumarate hydratase‐deficient cancer. Cancer Cell. 2014;26(6):840‐850. 25490448 10.1016/j.ccell.2014.10.005 PMC4386283

[28] Bradley WD , Arora S , Busby J , et al. EZH2 inhibitor efficacy in non‐Hodgkin's lymphoma does not require suppression of H3K27 monomethylation. Chem Biol. 2014;21(11):1463‐1475. 25457180 10.1016/j.chembiol.2014.09.017

[29] Carden MA , Smith S , Meany H , Yin H , Alazraki A , Rapkin LB . Platinum plus bortezomib for the treatment of pediatric renal medullary carcinoma: two cases. Pediatr Blood Cancer. 2017;64(7):e26402. 10.1002/pbc.26402 28052556

[30] Chou TC . Theoretical basis, experimental design, and computerized simulation of synergism and antagonism in drug combination studies. Pharmacol Rev. 2006;58(3):621‐681. 16968952 10.1124/pr.58.3.10

