# Supplementary material for: Novel renal medullary carcinoma cell lines, UOK353 and UOK360, provide preclinical tools to identify new therapeutic treatments
Source: Genes Chromosomes Cancer. 2020 Apr 17;59(8):472–83. doi: 10.1002/gcc.22847 (PMC7383978; doi:10.1002/gcc.22847)
Supplement: Supplementary file 1 — Data S1: Supporting Information [file GCC-59-472-s001.docx]

**Supporting Information**

**for**

**Novel renal medullary carcinoma cell lines, UOK353 and UOK360, provide preclinical tools to identify new therapeutic treatments**

Darmood Wei^1^, Youfeng Yang^1^, Christopher J. Ricketts^1^, Cathy D. Vocke^1^, Mark Ball^1^, Carole Sourbier^1^, Darawalee Wangsa^2^, Danny Wangsa^2^, Rajarshi Guha^3^, Xiaohu Zhang^3^, Kelli Wilson^3^, Lu Chen^3^, Paul S. Meltzer^2^, Thomas Ried^2^, Craig J Thomas^3^, Maria J. Merino^4^, and W. Marston Linehan^1^

^1^ Urologic Oncology Branch, Center for Cancer Research, National Cancer Institute, National Institutes of Health, Bethesda, Maryland.

^2^ Genetics Branch, Center for Cancer Research, National Cancer Institute, National Institutes of Health, Bethesda, Maryland.

^3^ Division of Preclinical Innovation, National Center for Advancing Translational Sciences, National Institutes of Health, Rockville, Maryland.

^4^ Laboratory of Pathology, National Cancer Institute, National Institutes of Health, Bethesda, Maryland.


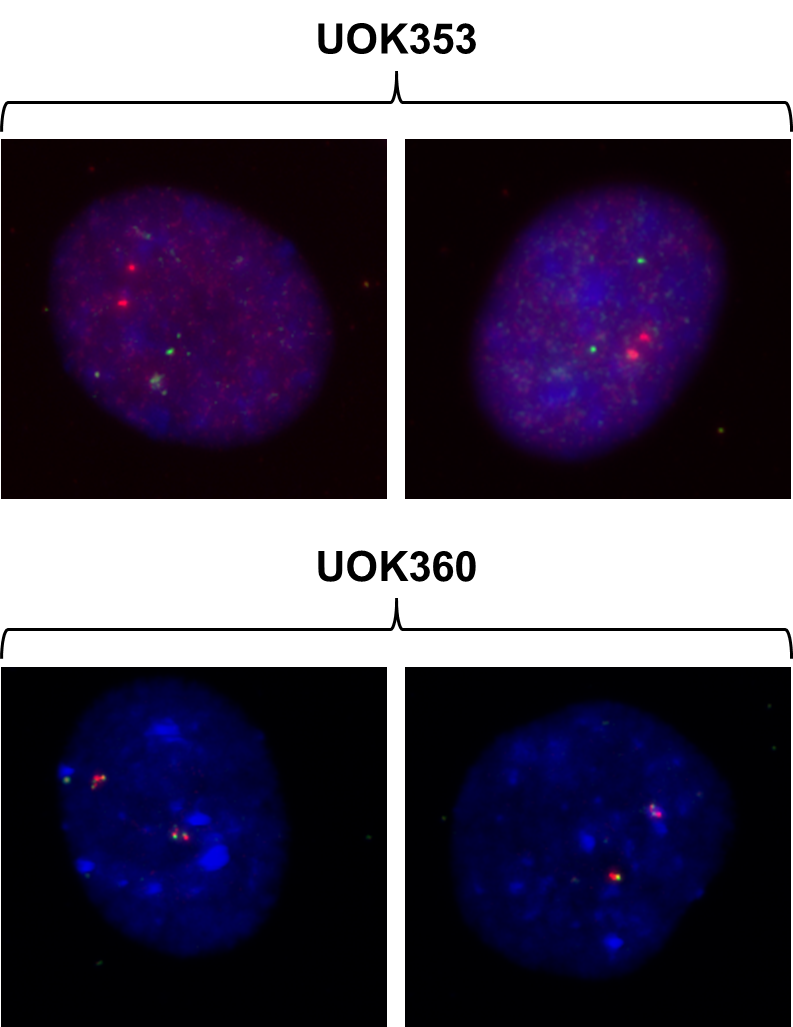


**Supporting Information Figure 1** FISH analysis of *SMARCB1* in UOK353 and UOK360.

SMARCB1 break apart probes were used to evaluate interphase spreads in both UOK353 and UOK360 and two example spreads are shown. The green probe is at the centromeric 5’ end of the *SMARCB1* gene and the red probe is at the telomeric 3’ end of the gene.


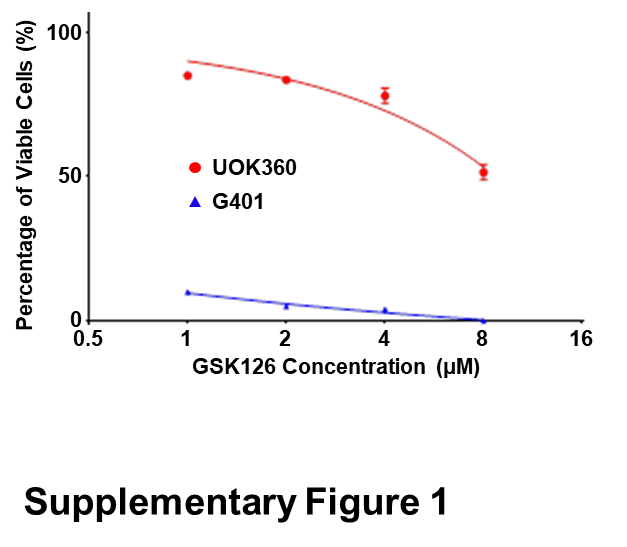


**Supporting Information Figure 2** EZH2 inhibitor of the malignant rhabdoid tumor cell line G401.

The sensitivity of UOK360 tumor spheroids to GSK126 was compared with the sensitivity of G401 tumor spheroids. UOK360 tumor spheroids were considerably more resistant than G401 to EZH2 inhibition.


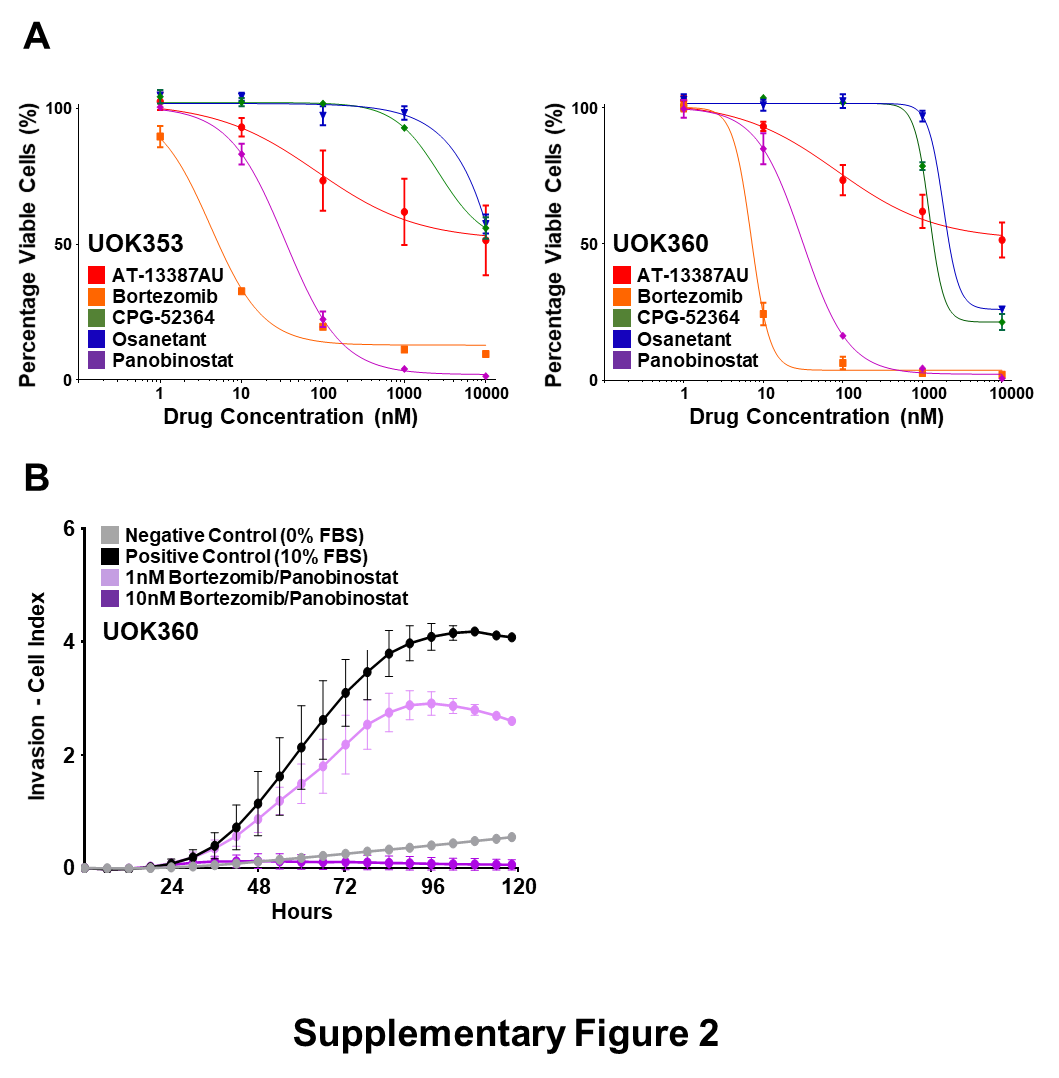


**Supporting Information Figure 3** Analysis of potential therapeutic agents in UOK360 and UOK353.

A, Confirmation assays demonstrated the IC50 concentrations for the potential therapeutic agents identified by high throughput drug screening, AT-13387AU, Bortezomib, CPG-52364, Osanetant, and panobinostat. B, Invasion assays using the xCELLigence system demonstrated that a combination of 1 nM of bortezomib and 1 nM panobinostat partially inhibited invasion of UOK360 but a combination 10 nM of bortezomib and 10 nM panobinostat almost completely inhibited invasion of UOK360 and not UOK353.


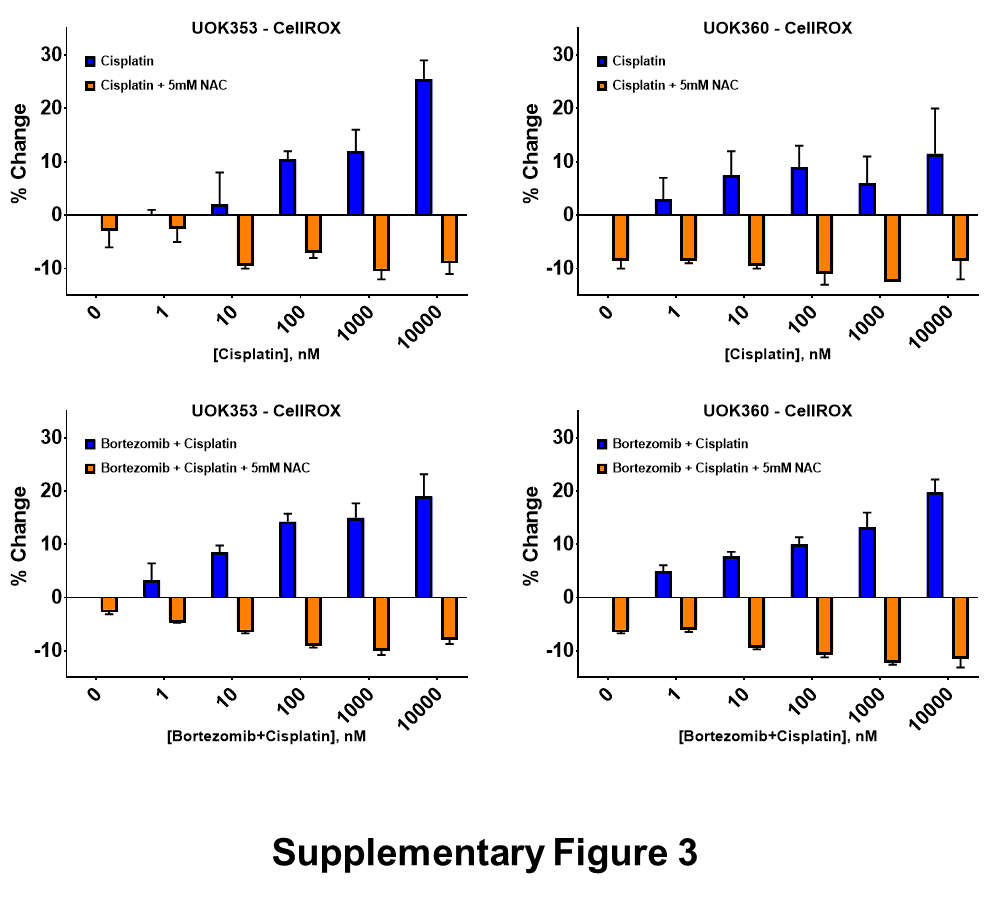


**Supporting Information Figure 4** Redox response to therapeutic treatments.

CellRox assays measured at 48 h post treatment demonstrated a dose dependent increase in redox in response to cisplatin and a combination of bortezomib and in both UOK353 and UOK360. All increases in redox in response to therapeutic agents were abrogated by the addition of 5 mM NAC at all concentrations in both cell lines.


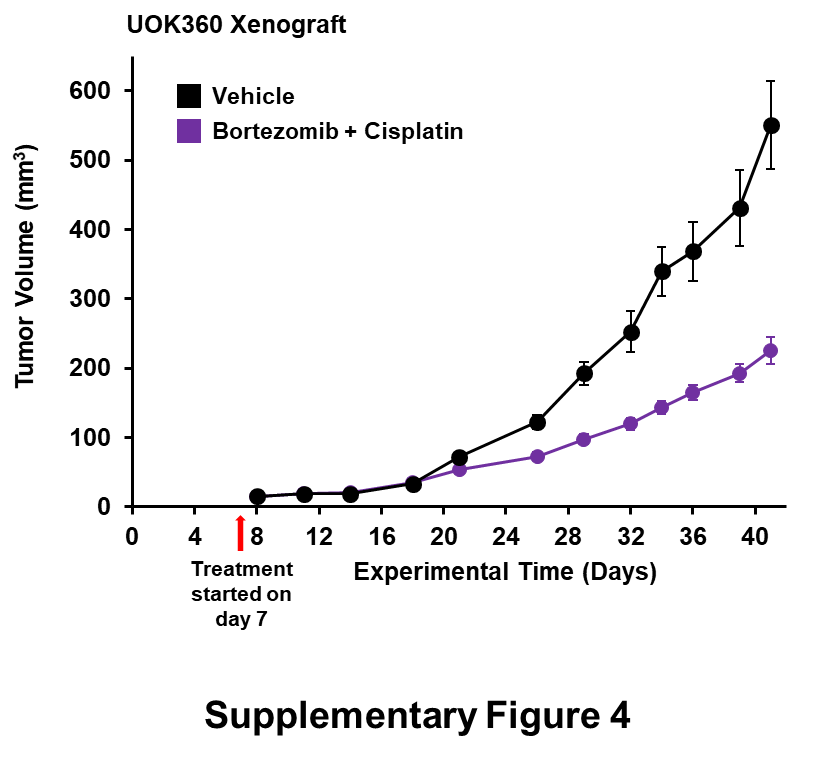


**Supporting Information Figure 5** Confirmation of combination therapy in UOK360 xenografts.

Mice were injected with 1 million UOK360 xenograft tumors and treated by intraperitoneal injection twice weekly with either 0.9% saline vehicle or a combination of bortezomib and cisplatin (n=13) and followed for 6 weeks. Treatment was started 7 days after the initial injection and tumors were measured from day 8 onwards.
